# Impact of a moderate-intensity aerobic exercise intervention on systemic and uterine natural killer cells in women with unexplained recurrent pregnancy loss

**DOI:** 10.3389/fimmu.2025.1602939

**Published:** 2025-06-04

**Authors:** Aysel Gurbanova, Amber E. M. Lombardi, Denise H. J. Habets, Salwan Al-Nasiry, Marc E. A. Spaanderman, Marien I. de Jonge, Tess Meuleman, Lotte Wieten, Renate G. van der Molen

**Affiliations:** ^1^ Department of Laboratory Medicine, Laboratory of Medical Immunology, Radboud University Medical Center, Nijmegen, Netherlands; ^2^ Department of Transplantation Immunology, Maastricht University Medical Center+, Maastricht, Netherlands; ^3^ GROW – Research Institute for Oncology and Reproduction, Maastricht University, Maastricht, Netherlands; ^4^ Department of Obstetrics and Gynecology, Maastricht University Medical Center+, Maastricht, Netherlands; ^5^ Department of Obstetrics and Gynecology, Radboud University Medical Center, Nijmegen, Netherlands

**Keywords:** NK cells, exercise intervention, recurrent pregnancy loss (RPL), menstrual blood, immunophenotyping

## Abstract

**Introduction:**

Recurrent pregnancy loss (RPL) is associated with altered immune phenotypes and functions. It has been proposed that physical exercise might impact the immune system. Therefore, we evaluated the effect of a personalized 3-month moderate-intensity aerobic exercise intervention on the immune system of women with unexplained RPL (uRPL). Given the suggested supportive role of Natural Killer (NK) cells during early pregnancy, we focused on numerical, phenotypic, and functional changes in peripheral NK (pNK) and uterine NK (uNK) cells.

**Methods:**

Mononuclear cells were isolated from peripheral blood (PB) (n=23) and menstrual blood (MB) (n=22) of women with uRPL. NK cell phenotypes were assessed with comprehensive flow cytometry panels. NK cell function was assessed with degranulation assays and intracellular staining of interferon-γ (IFN-γ), perforin and granzyme-B in a subgroup of women due to lower availability of samples (n=12).

**Results:**

Attendance to the exercise intervention was overall 95%, which resulted in effects on the phenotype and function of pNK cells. We found a significant reduction in the median fluorescent intensity of CD161 (464 *vs* 410, p=0.011), NKp30 (432 *vs* 376, p=0.018), and NKG2A (886 *vs* 732, p=0.039) in pNK cells after exercise, while no differences were observed in uNK cells. We also observed decreased percentages of IFN-γ^+^ pNK cells (49% *vs* 25.2%, p=0.027) after exercise.

**Discussion:**

Our study shows promising results, suggesting that exercise can impact pNK cell phenotype and function in women with uRPL. Following the changes in pNK phenotype and function suggest a lower pro-inflammatory state post-exercise. Whether these exercise-induced phenotypic and functional changes of pNK cells impact subsequent pregnancies remains to be studied. The study details are available through HYPERLINK “https://clinicaltrials.gov/”Home | ClinicalTrials.gov, trial ID: HMOVE

## 1 Introduction

Recurrent pregnancy loss (RPL) is a pregnancy disorder affecting 1-2% of women attempting to conceive and is defined by the failure of two or more clinically recognized pregnancies before 24 weeks of gestation (1). The etiologies for RPL include advanced maternal age, abnormal parental karyotyping (if indicated), endocrine disorders, uterine abnormalities, antiphospholipid syndrome, lifestyle factors, and embryonic factors (1). In addition, women with RPL have a higher incidence of metabolic disorders (2–4), and an elevated risk of developing cardiovascular disease (5–7). However, in approximately 50% of cases, the underlying cause remains unknown, referred to as “unexplained” RPL (uRPL) (8).

It is well-recognized that the maternal immune system plays a critical role in the establishment and maintenance of pregnancy (9, 10). During early pregnancy, 70% of all infiltrating leukocytes are uterine Natural Killer (uNK) cells (11). They are primarily CD56^bright^CD16^-^ and exhibit functional and phenotypic differences compared to peripheral NK (pNK) cells, which are predominantly CD56^dim^CD16^+^ (12). It is known that uNK cells are involved in regulating trophoblast invasion and spiral artery remodeling (13–16). Numerous studies have investigated the role of NK cells in the etiology of RPL by examining uterine tissue and peripheral blood (PB) (15, 17–19). Some of these studies reported altered numbers and percentages of NK cells in the endometrium and decidua (20, 21) of women with RPL compared to controls. Additionally, several studies have demonstrated altered NK cell receptor expression (20, 22–25) and increased NK cell cytotoxicity in women with RPL (26–28). However, there are also studies indicating no changes in NK cells of women with RPL (29, 30).

Multiple studies have shown that physical exercise can modulate the immune system and influence the distribution, phenotype, and activity of different immune cell subsets, as summarized in various reviews (31–33). NK cells seem to be particularly responsive to exercise, acutely mobilizing into the circulation immediately after exercise (34–36). Notably, this mobilization primarily involves more mature and differentiated NKG2A^-^/Killer Immunoglobulin-like (KIR)^+^ NK cells compared to the less mature NKG2A^+^/KIR^-^ NK cells in PB (37). It is hypothesized that after rapid mobilization to the bloodstream NK cells redistribute to peripheral tissues, as a drop in circulating NK cells to baseline levels was observed 2 hours after acute exercise in men (38). Moreover, a recent meta-analysis showed that the cytolytic activity of NK cells increases after acute exercise (39). However, the effects of exercise on tissue-resident immune cells, such as those in the uterine environment, remain understudied.

Considering altered numbers, phenotypes and function of NK cells have been reported in uRPL, we aimed to investigate the potential effects of a moderate-intensity aerobe exercise intervention on NK cell percentages, phenotype and function. Moreover, we have also investigated the impact of exercise on different T- and B-cell population frequencies. In this feasibility study, we focused on the impact of a three-month moderate-intensity aerobic exercise intervention on both peripheral immune cells and uterine immune cells by sampling menstrual blood (MB) as source of uterine-derived immune cells (40).

## 2 Materials and methods

### 2.1 Study population

In this multi-center intervention study, 49 women with uRPL were included (**Supplementary Figure 1**). uRPL was defined as having two or more pregnancy losses before 24 weeks of gestation without an identifiable cause. Women were referred to the department of Obstetrics and Gynecology at either the Radboud University Medical Centre (RUMC) or the Maastricht University Medical Centre+ (MUMC+) and were included when no cause for their miscarriages could be identified according to the guidelines of the European Society of Human Reproduction and Embryology (ESHRE). The clinical work-up included a standardized medical history of the couple, screening for uterine abnormalities, thyroid abnormalities, anti-phospholipid syndrome, paternal screening for sperm DNA fragmentation and parental karyotyping (if indicated) (41). Participants were excluded from the study if they met one or more of the exclusion criteria presented in **Supplementary Table 1**.

This study was conducted in accordance with the Declaration of Helsinki, the Medical Research Involving Human Subjects Act, and the guidelines for Good Clinical Practice (GCP). After obtaining informed consent, according to the Medical Ethical Committee of the Radboud University Medical Centre and Maastricht University Medical Centre+ NL77307.091.21, women received a questionnaire for assessing baseline characteristics. PB and MB were collected for immunophenotyping.

The intervention consisted of a 12-week, personalized, moderate-intensity, aerobe exercise program for a stationary bike. The exercise program was personalized to individual heart rate reserves (HRR). The first six weeks the participants were instructed to train two times a week for 50 minutes at 50-60% of their HRR, the subsequent six weeks for three times a week for one 50 minutes at 50-60% HRR. Their heart rate was monitored during exercise using a Polar H10 heartrate monitor chest strap (Polar Electro, Inc, Kempele Finland). Women needed to attend a minimum of 80% of 30 prescribed trainings after completion of the intervention in order to be eligible for data analysis. All women were asked not to change their pre-existing training schedules (if applicable) and not to change their diet during the intervention. Before and after the 12-week exercise intervention, identical immune analyses were conducted.

Of the 49 women who were included in the study, 24 women completed the intervention (data is shown only of these women) (**Table 1**). Both PB and MB samples were collected. However, due to collection issues, PB was available from 23 participants, and MB was available from 22 participants. Different figures might show variable numbers of women included in analyses if unreliable individual markers were excluded due to technical staining issues. Sufficient remaining cells (n=12) were frozen for later functional analyses.

**Table 1:** Baseline characteristics of the study population.

Characteristic	Intervention (n=24)
Age (years)	35.0 (30.5-38.0)
BMI (kg/m^2^)	25.0 (21.7-28.2)
Chronic disease	16.7%
	IgA nephropathy (1/24) Congenital hypothyroidism (1/24) Polycystic Ovary Syndrome (1/24) Celiac disease (1/24)
Medication use	20.8%
	Thyromimetics (3/24) Anti-depressants (1/24) Acetylsalicylic acid (1/24)
Smoking	
- Current - Former - Non-smoker	8.3% 20.8% 70.8%
Alcohol use	50%
Regular menstrual cycle	79.2%
Gravida	5.5 (4.3-7.8)
Para	0.0 (0.0-1.0)
Pregnancy losses	4.0 (3.3-7.0)
- Primary RPL	54.2%
- Secondary RPL	45.8%
- Early miscarriage (<10 weeks)	4.0 (2.0-7.0)
- Late miscarriage (≥10 weeks)	0.0 (0.0-1.0)

Medication use means the percentage of people that are currently using medication. Alcohol use means the percentage of people that consume at least one glass of alcohol/week. Gravida is number of pregnancies, para is the number of live births. Data are presented as either a percentage (chronic disease, medication use, smoking, alcohol use, regular menstrual cycle, and primary vs secondary RPL), or as a median with interquartile range (age, BMI, gravida, para, pregnancy losses, early miscarriage, and late miscarriage).

### 2.2 Isolation of menstrual blood mononuclear cells and peripheral blood mononuclear cells

To study uterine lymphocytes, MB was collected using a menstrual cup (40) at 3 time points of 12 hours right after the start of menses, for a total of 36 hours. After every 12 hours, the cup was emptied in a new collection tube, containing RPMI1640 medium (ThermoFisher Scientific, Waltham, MA, USA) supplemented with 10% human pooled serum (HPS) (Zen-Bio, Durham, NC, USA), 1 mM pyruvate (ThermoFisher Scientific), 2 mM GlutaMAX™ (ThermoFisher Scientific), 100 U/mL penicilin/100 μg/mL streptomycin (ThermoFisher Scientific), and 0.3% sodium citrate (Merck, Darmstadt, Germany). PB (10 mL) was collected in ethylene diamine tetra acetic acid (EDTA) tubes.

PB was diluted 1:1 with phosphate buffered saline (PBS) (Fresenius Kabi, GmbH, Graz, Austria). The collected MB from 3 different timepoints was first pooled, after which it was washed with PBS. After centrifugation, the pellet of the MB was filtered through a 70 μm cell strainer (PluriSelect Life Science, Leipzig, Germany) by creating a vacuum using a connector ring (PluriSelect) and a 30 mL syringe (Terumo Corporation, Tokyo, Japan). The filtered MB was then incubated for 20 minutes at room temperature with RosetteSep™ Human Granulocyte Depletion Cocktail (STEMCELL Technologies, Vancouver, Canada) according to manufacturer’s instructions, after which the sample was diluted with an equal volume of PBS+2%HPS.

Subsequently, MB mononuclear cells (MBMCs) and PB mononuclear cells (PBMCs) were isolated using Lymphoprep (STEMCELL Technologies) according to manufacturer’s instructions. The samples were centrifuged for 15 min at 800g, no brake at 20°C. Isolated MBMCs and PBMCs were washed twice with PBS+2%HPS, and resuspended in RPMI1640 + 10% HPS.

### 2.3 Antibodies and flow cytometry analyses

Immunophenotyping of MBMCs and PBMCs was done using flow cytometry. For the phenotyping, 500.000 MBMCs and 250.000 PBMCs were used and stained in 50 μL and 25 μL respectively. First, the cells were stained for 30 minutes at 4°C with eBioscience™ Fixable Viability Dye eFluor 780 (ThermoFisher Scientific) to stain for dead cells. Subsequently, the cells were stained for 30 minutes at 4°C for specific surface markers stated in **Supplementary Table 2**. Because of the use of multiple BD Horizon™ Brilliant Violet Dyes (BD, Franklin Lakes, NJ, USA), BD Horizon™ Brilliant Staining Buffer (BD) was used in combination with regular FACS buffer (PBS+1%BSA+0.02% Sodium Azide (Merck, Rahway, NJ, USA)) according to manufacturer’s recommendations. After surface staining, all cells were fixated and permeabilized using the eBioscience™ Foxp3/Transcription Factor Staining Buffer Set according to the manufacturer’s instructions (ThermoFisher Scientific). After fixation/permeabilization, part of the cells were stained for the transcription factor FoxP3 (ThermoFisher Scientific). All samples were measured with standardized settings on the BD FACSLyric™ (BD). Data was analyzed using the FlowJo™ v10.8 software (Flowjo™, Ashland, OR, USA) as either a percentage or as Median Fluorescent Intensity (MFI). MFIs of immune markers were normalized against MFIs of Fluorescence Minus One (FMO) controls, critical to ensure that the signals observed are truly due to the marker of interest and not artefacts from overlapping fluorescent markers. Certain markers for specific signals, timepoints or participants could not be analyzed due to various reasons such as sample or reagent availability. PBMCs and MBMCs that were not directly used in the flow cytometry staining were frozen in Recovery™ Cell Culture Freezing Medium (ThermoFisher Scientific).

### 2.4 Degranulation assay and staining of intracellular molecules

To assess the functional capacity of NK cells in MB and PB, we performed a CD107a degranulation assay and an intracellular staining on frozen PBMCs and MBMCs. Following thawing of a subgroup of available samples (n=12), 200,000 cells were plated in a round-bottom 96-wells plate. Cells that were used to investigate CD107a and interferon-γ (IFN-γ) expression were stimulated with 100 U/mL IL-2 and 10 ng/mL IL-15. An unstimulated condition was taken as a control. The cells that were used to investigate granzyme B and perforin expression were left unstimulated. The cells were incubated at 37°C and 5% CO_2_. After 20 hours of incubation, 25 μL of a 1:50 dilution of CD107a-PE (BD Biosciences) was added to the respective wells. One hour later, 20,000 K562 target cells and 5 μg/mL of Brefeldin A and 5 μg/mL of Monensin were added to inhibit the internalization of CD107a molecules and enhance the detection of intracellular proteins. The cells in the wells for investigation of granzyme B and perforin expression were not stimulated with K562. The cells were then incubated for an additional 3 hours at 37°C in 5% CO_2_. Following the incubation period, cell viability was assessed, and surface markers were stained with the following antibodies: anti-CD16 Alexa Fluor 700 (BioLegend), anti-CD45 Krome Orange (Beckman Coulter), anti-CD3 BV605 (BD Biosciences), and anti-CD56 BV711 (BD Biosciences). Subsequently, intracellular staining was performed using anti-granzyme B PerCP-Cy5.5 (BioLegend), anti-IFNγ PE-Cy7 (ThermoFisher Scientific), and anti-perforin Pacific Blue (BioLegend) (**Supplementary Table 2**). All samples were analyzed on a BD FACSLyric™ flow cytometer (BD Biosciences).

### 2.5 Statistics

Percentages of immune cells or percentages/MFIs of immune markers before and after exercise were analyzed with a Wilcoxon signed-rank test. All analyses were performed using IBM SPSS statistics, version 29.0.0.0 (SPSS Inc, Chicago, IL, USA) and P-values <0.05 were considered statistically significant. The boxplots were prepared in RStudio using ggplot2 package (42, 43).

## 3 Results

### 3.1 Moderate-intensity aerobic exercise does not alter immune cell frequencies in MB and PB of women with uRPL

**Figure 1:**
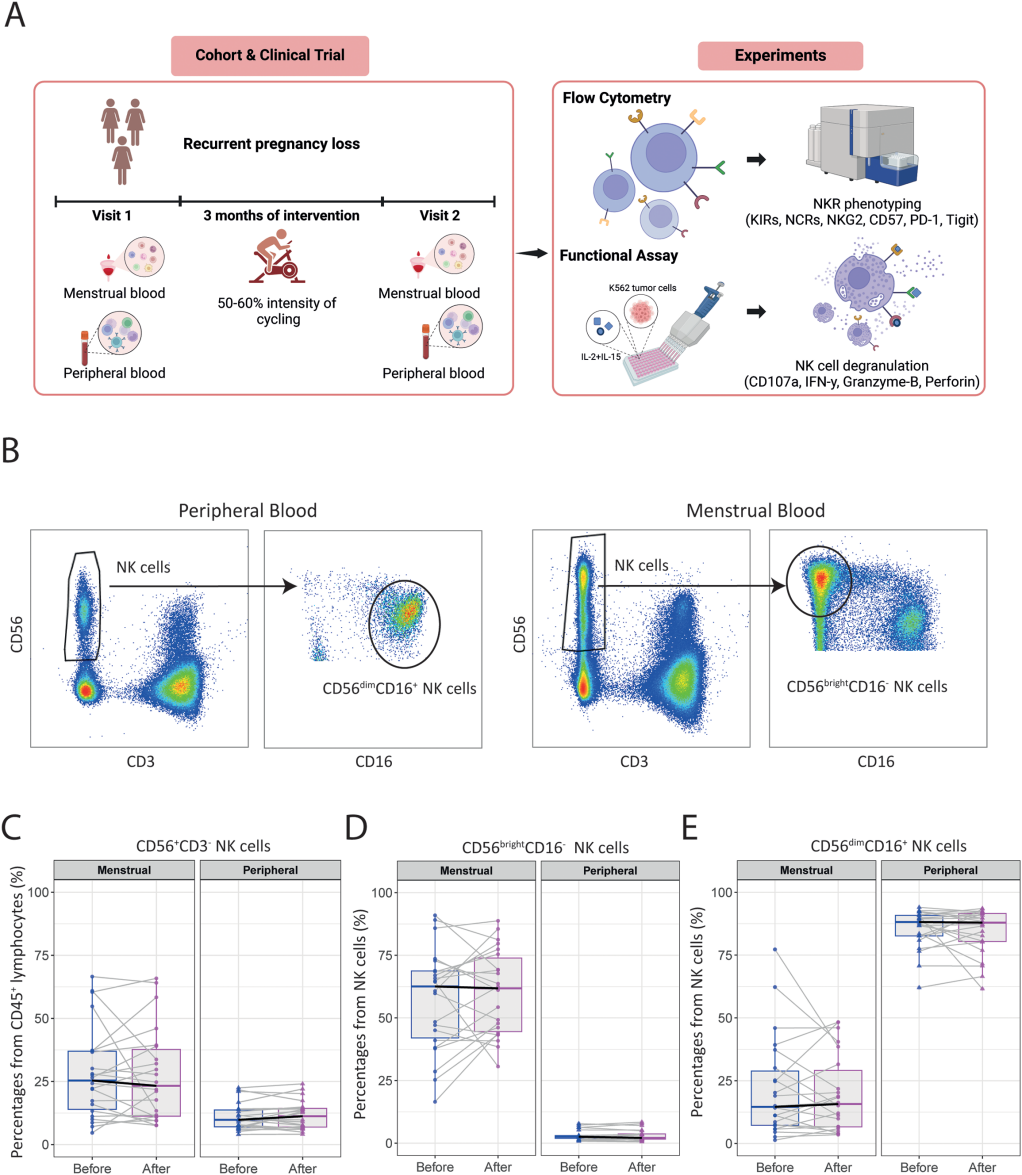
The moderate-intensity aerobic exercise intervention does not alter immune cell frequencies in MB or PB of women with uRPL. **(A)** Overview of the study and the experiments performed on the isolated immune cells. **(B)** Representative dot plots showing the gating strategies for NK cell subsets in PB and MB. **(C)** The percentage of total CD56^+^CD3^-^ NK cells from CD45^+^ lymphocytes in MB and PB before and after the moderate-intensity aerobic exercise intervention. **(D)** The percentage of CD56^bright^CD16^-^ NK cells of total NK cells. **(E)** The percentage of CD56^dim^CD16^+^ NK cells of total NK cells. PB n=23, MB n=22. The dots represent the uterine NK cells, the triangles the peripheral NK cells (blue=before intervention, magenta=after intervention). Boxplots visualize the median (bold black line) and the interquartile ranges. A Paired Wilcoxon signed-rank test was used to determine statistical significance.

To determine the effect of exercise on specific immune cell subsets, we analyzed frequencies of T, B, and NK cells using high-dimensional flow cytometry in both PB and MB from women with uRPL (**Figure 1A**). Baseline characteristics, of the included 24 women who completed the intervention with an overall attendance of 95%, are shown in **Table 1**.

The role of NK cells in PB and MB are different. While the majority of pNK cells consist of a subset of CD56^dim^CD16^+^ NK cells with high cytotoxic capabilities, uNK cells in MB mainly have a CD56^bright^CD16^-^ phenotype and a immunoregulatory functions (12). Results of these two subsets (**Figure 1B**) revealed no significant differences in the frequency of total CD56^+^CD3^-^, CD56^bright^CD16^-^ and CD56^dim^CD16^+^ NK cells after intervention in either PB or MB (**Figures 1C–E**). Interestingly, we observed significant heterogeneity in the baseline frequencies of uNK cells among women with uRPL, as well as in their response to the intervention. While some women exhibited increased frequencies of uNK cells, others showed a decrease post-intervention.

Likewise, the frequencies of total CD3^+^ T cells and their subsets (including conventional effector CD4^+^, cytotoxic CD8^+^ T, and CD127^low^CD25^+^FoxP3^+^ regulatory T cells) remained similar in both PB and MB when comparing pre- and post-intervention measurements (**Supplementary Figures 2A–C**). Although there was a trend towards an increased frequency of total CD19^+^CD20^+^ B cells in PB as well as MB, the frequencies of B cell subsets did not change significantly after intervention (**Supplementary Figures 2D–F**).

### 3.2 CD161 (KLRB1) and NKp30 expression by pNK cells is decreased after a moderate-intensity aerobic exercise intervention in women with uRPL

To study the effect of the intervention on the phenotype of pNK and uNK cells, we performed comprehensive immunophenotyping by flow cytometry to evaluate changes in activating and inhibitory NK cell receptors (NKRs), frequently described to be involved in the regulation of NK cell activity.

**Figure 2:**
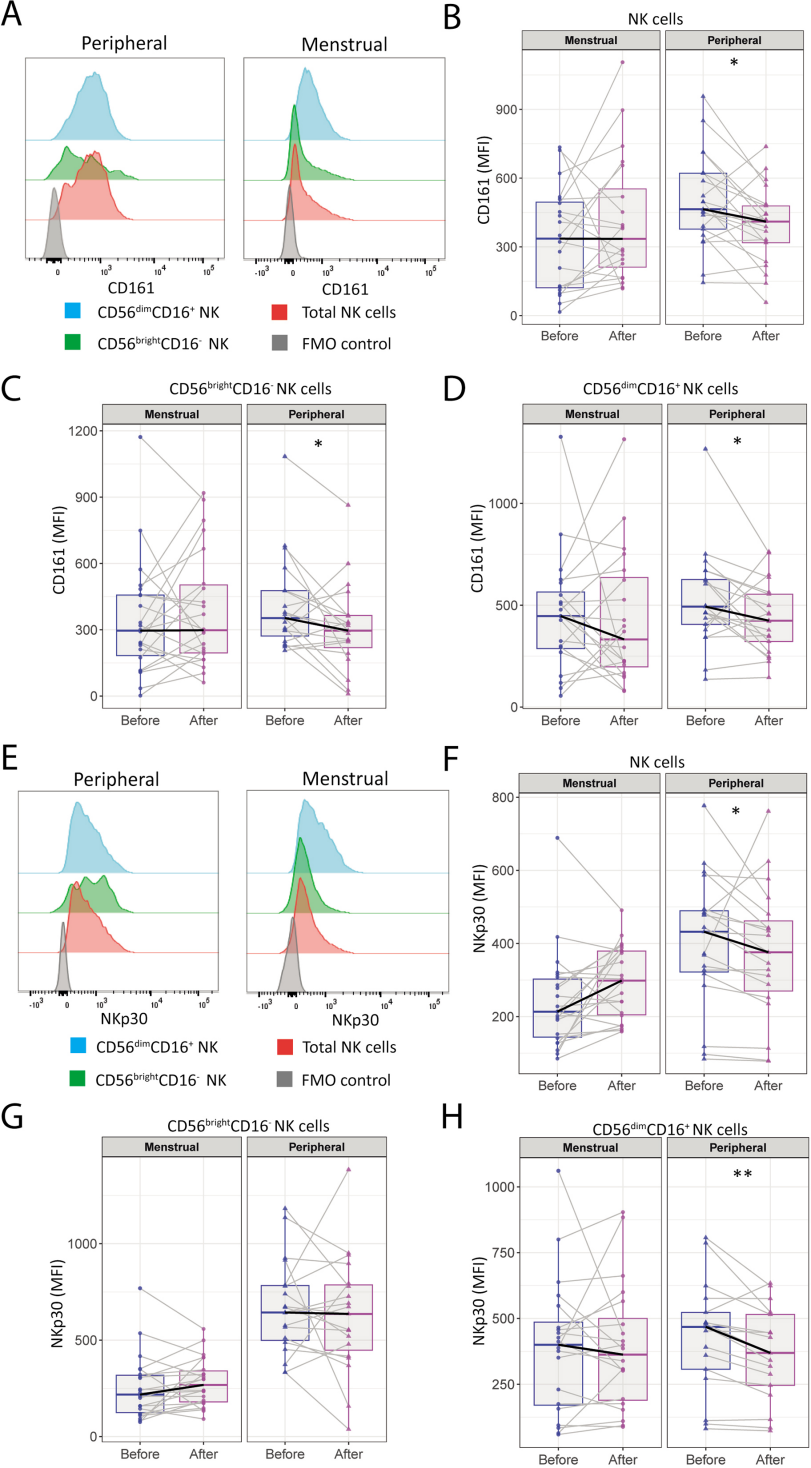
CD161 (KLRB1) and NKp30 expression by peripheral NK cells is decreased after the moderate-intensity aerobic exercise intervention in women with uRPL. **(A)** Representative histograms of CD161 expression by total NK cells (red), CD56^dim^CD16^+^ NK cells (blue), CD56^bright^CD16^-^ NK cells (green), and the fluorescence minus one (FMO) control (gray) of MB and PB. **(B)** Median fluorescent intensity (MFI) of CD161 by total uterine NK (uNK) and peripheral NK (pNK) cells before and after the moderate-intensity aerobic exercise intervention. **(C)** MFI of CD161 by CD56^bright^CD16^-^ NK cells. **(D)** MFI of CD161 by CD56^dim^CD16^+^ NK cells. **(E)** Representative histograms of NKp30 expression by total NK cells (red), CD56^dim^CD16^+^ NK cells (blue), CD56^bright^CD16^-^ NK cells (green), and the FMO control (gray) of MB and PB. **(F)** MFI of NKp30 by NK cells. **(G)** MFI of NKp30 by CD56^bright^CD16^-^ NK cells. **(H)** MFI of NKp30 by CD56^dim^CD16^+^ NK cells. PB n=21, MB n=22. All MFIs were normalized to the MFI of the FMO control. The dots represent the uNK cells, the triangles the pNK cells (blue=before intervention, magenta=after intervention). Boxplots visualize the median (bold black line) and the interquartile ranges. A Paired Wilcoxon signed-rank test was performed to determine statistical significance (*p<0.05, **p<0.005).

We found a significant decrease in median fluorescent intensity (MFI) of CD161 (KLRB1) in total pNK cells (464 [364-623] to 410 [277-483]; p=0.011), in the CD56^bright^CD16^-^ pNK cell subset (353 [247-478] to 296 [193-367]; p=0.033), and in the CD56^dim^CD16^+^ pNK cell subset (493 [393-648] to 424 [296-559]; p=0.035), while no significant differences were observed in uNK cells post-intervention (**Figures 2A–D**).

Among the natural cytotoxicity receptors (NCRs) analyzed, the MFI of NKp30 significantly decreased in total pNK cells (432 [317-494] to 376 [252-481]; p=0.018) and in the CD56^dim^CD16^+^ pNK subset (468 [290-550] to 369 [228-520]; p=0.004) post-intervention, whereas this was not observed in uNK cells (**Figures 2E–H**). No significant changes were found in the expression intensities of other NCRs (NKp44 and NKp46) after intervention (**Supplementary Figures 3A, B**).

### 3.3 Moderate-intensity aerobic exercise impacts NKG2A and NKG2C expression by pNK cells in women with uRPL

**Figure 3:**
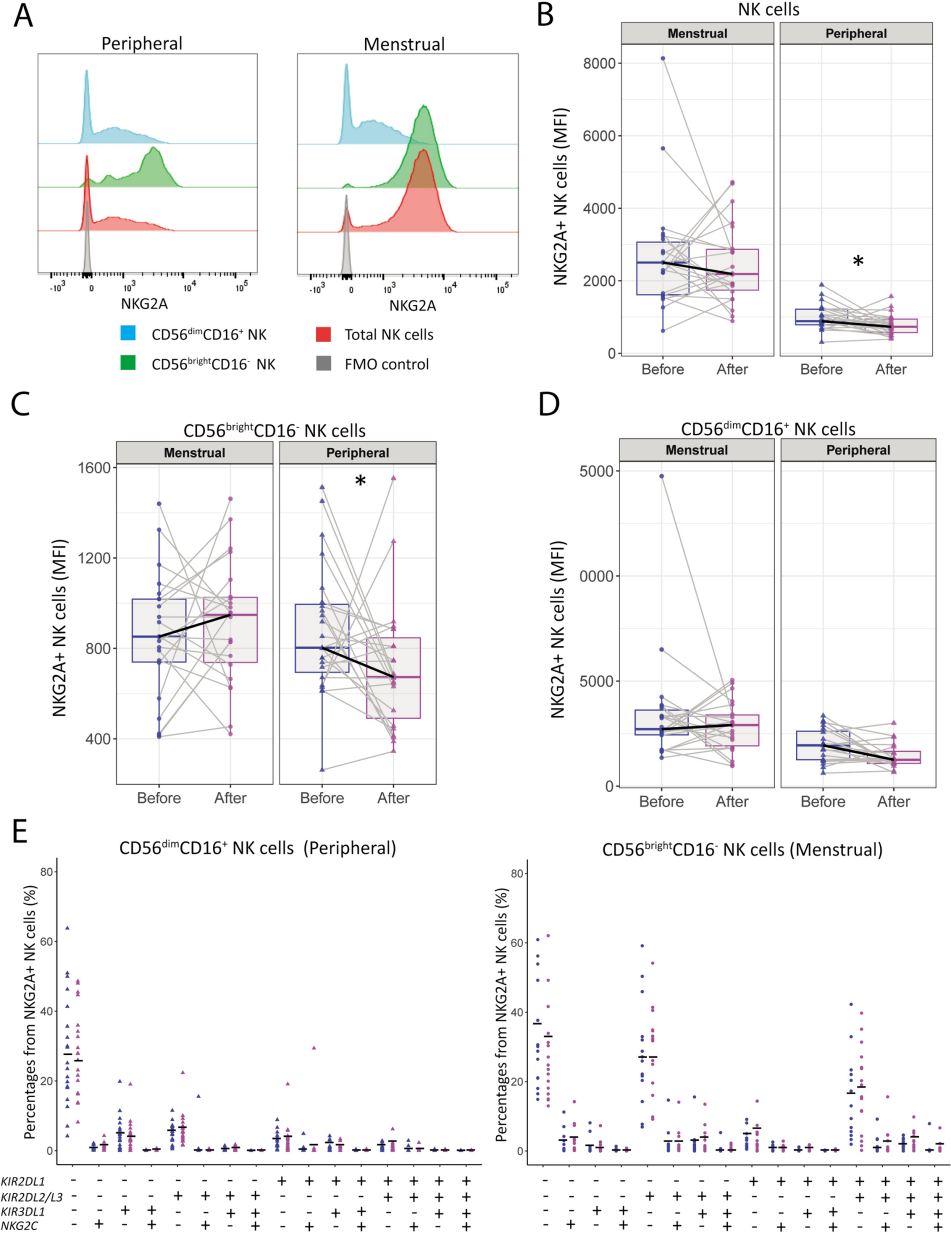
NKG2A expression by peripheral NK is decreased after the moderate-intensity aerobic exercise intervention in women with uRPL. **(A)** Representative histograms of NKG2A expression by total NK cells (red), CD56^Dim^CD16^+^ NK cells (blue), CD56^bright^CD16^-^ NK cells (green), and the fluorescence minus one (FMO) control (gray) of MB and PB. **(B)** Median fluorescent intensity (MFI) of NKG2A by total NK cells in MB and PB before and after the moderate-intensity aerobic exercise intervention. **(C)** MFI of NKG2A by CD56^bright^CD16^-^ NK cells. **(D)** MFI of NKG2A by CD56^dim^CD16^+^ NK cells. PB n=23, MB n=22. **(E)** Tree plots showing percentages of NK cell clusters positive for NKRs (*KIR2DL1, KIR2DL2/L3, KIR3DL1, NKG2C*) in NKG2A^+^ CD56^dim^CD16^+^ peripheral NK (pNK) cells and CD56^bright^CD16^-^ uterine NK (uNK) cells (PB n=18, MB n=16). MFIs were normalized to the MFI of the FMO control. The dots represent the uNK cells, the triangles the pNK cells (blue=before intervention, magenta=after intervention). Boxplots visualize the median (bold black line) and the interquartile ranges. A Paired Wilcoxon signed-rank test was performed to determine statistical significance (*p<0.05).

To study the effect of exercise on NK cell markers involved in maturation and memory, we analyzed the frequency and expression intensity of NKG2A and NKG2C before and after intervention. While the frequencies of NKG2A^+^ NK cells did not significantly change after intervention in either MB or PB (**Supplementary Figures 4A–C**), a marked decrease in the MFI of NKG2A was observed in total pNK cells (886 [781-1218] to 732 [555-962]; p=0.039) and CD56^bright^CD16^+^ pNK cells (2472 [1583-3116] to 1497 [1113-1790]; p=0.034) (**Figures 3A–C**). In contrast, uNK cells did not show any significant changes in NKG2A expression (**Figures 3A–D**). Furthermore, no significant changes were observed in both frequency and expression intensity of NKG2C in both MB and PB, except for a slight increase the percentage of in NKG2C^+^ CD56^dim^CD16^+^ pNK cells (5.4 [3.3-13.7] to 5.9 [2-8.9]; p=0.021) (**Supplementary Figure 4F**).

Next we investigated co-expression of NKRs by analyzing the expression of NKG2A, NKG2C, and KIRs, on CD56^dim^CD16^+^ pNK cells and CD56^bright^CD16^-^ uNK cells. No significant changes were found in the CD56^dim^CD16^+^ pNK cells or CD56^bright^CD16^-^ uNK cells populations (**Figure 3E**). Additionally, the frequencies of CD57 and KIRs remained unchanged (**Supplementary Figures 3C–F**).

### 3.4 Exercise reduces the IFN-γ production by pNK cells in uRPL women

**Figure 4:**
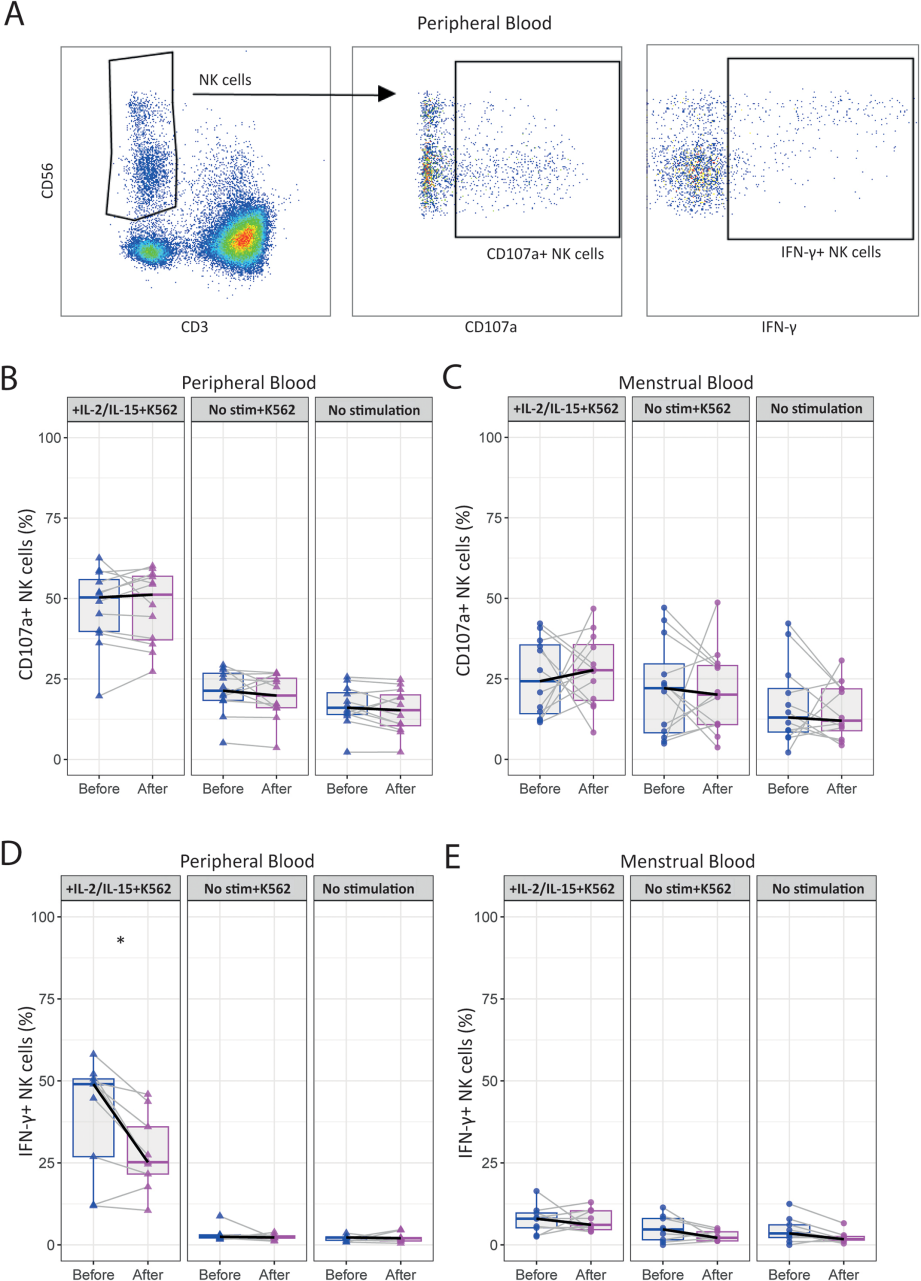
The moderate-intensity aerobic exercise intervention reduces the IFN-γ production by peripheral NK cells in women with uRPL. **(A)** Representative dot plots showing the gating strategy for determining IFN-γ^+^ and CD107a^+^ peripheral NK (pNK) cells after 24h of stimulation with IL-2 and IL-15 and a 4h co-culture with K562 tumor cells. **(B)** Percentages of CD107a^+^ pNK cells stimulated with IL-2 and IL-15, and co-cultured with K562 (IL-2/IL-15+K562, left panel), only co-cultured with K562 (No stim+K562, central panel), and unstimulated (No stimulation, right panel) conditions. **(C)** Percentages of CD107a^+^ uterine NK (uNK) cells. **(D)** Percentages of IFN-γ^+^ pNK cells. **(E)** Percentages of IFN-γ^+^ uNK cells. PB n=12, MB n=12. The dots represent the uNK cells, the triangles the pNK cells (blue=before intervention, magenta=after intervention). Boxplots visualize the median (bold black line) and the interquartile ranges. A Paired Wilcoxon signed-rank test was used for statistical significance (*p<0.05).

Lastly, we investigated the effect of the intervention on NK effector function by determining the levels of intracellular granzyme B and perforin. In addition, we analyzed NK cell degranulation (CD107a) and IFN-γ production upon stimulation with K562 target cells or cytokines (IL-2 and IL-15) before and after intervention. Our analysis revealed no significant changes in CD107a expression intensity or frequency in either pNK or uNK cells after intervention (**Figures 4B, C**, **Supplementary Figure 5A**). Notably, stimulation with K562 cells did not enhance CD107a expression in uNK cells compared to the unstimulated conditions (**Figure 4C**). IFN-γ production was significantly reduced by pNK cells (35.8 [3.5-50.4] to 23.1 [3.2-33.9]; p=0.028) after intervention, with no substantial change observed in uNK cells (**Figures 4D, E**). Overall, uNK cells demonstrated lower IFN-γ production compared to their peripheral counterparts upon stimulation and co-culture. Granzyme B and perforin production remained unchanged post-intervention in unstimulated pNK cells (**Supplementary Figures 4B, C**).

## 4 Discussion

This study investigated the effects of a moderate-intensity aerobic exercise intervention on the immune profiles of women with uRPL, focusing on immune cell frequencies, NK cell receptor repertoire, and NK cell functional responses. To our knowledge, this is the first study investigating the impact of an exercise intervention strategy on systemic (PB) and uterine (MB) immune cells, particularly in women with uRPL. The use of MB, a less commonly studied but highly relevant derivate of uterine tissue in reproductive research, can aid in providing better understanding whether exercise could impact the uterine immune environment. By conducting comprehensive analyses of NK cell receptor repertoires and functional degranulation assays in both PB and MB, we aim to provide new insights into characterization of potential immunomodulatory effects of exercise in women with uRPL.

The moderate-intensity aerobic exercise intervention was shown to induce phenotypic and functional changes in pNK cells. Firstly, we found a significant decrease in the expression of NKp30 by pNK cells after intervention, while no significant changes were detected in uNK cells. NKp30 is a critical activating receptor expressed on both mature resting and activated NK cells (44). Depending on the isoform, NKp30 can either enhance NK cell activation or promote the secretion of immunosuppressive cytokines, such as IL-10 (45). In the context of uRPL, some studies have reported a higher expression of NKp30 compared to healthy controls (46, 47), however, others have reported no significant differences (24, 25). In addition to a decreased expression of NKp30, we also found a reduced expression of CD161 (KLRB1) by pNK cells after exercise. Previously, Ntrivalas et al. investigated CD161 expression in uRPL patients and healthy controls but did not find any differences (48). It has been shown that high CD161 expression on NK cells correlates with pro-inflammatory NK cells indicating high cytokine responsiveness (49, 50). Crosslinking of CD161 with its ligand lectin-like transcript 1 (LLT1) can increase IFN-γ expression (49, 51). Indeed, our functional assay revealed decreased IFN-γ production by pNK cells following exercise suggesting potential association with decreased expression of CD161. In RPL patients, higher levels of IFN-γ have been found compared to controls, and this increase has been positively correlated with NK cell levels (52). However, it is important to note that only a small proportion of samples could be used in the functional assays. This reduces the ability to detect more subtle effects. In addition, we found significant decrease in expression intensity of NKG2A in pNK cells following intervention. Exercise has been shown to preferentially mobilize more mature and differentiated NKG2A^-^/KIR^+^ NK cells in PB (37). While we did not observe changes in the frequencies of KIRs after exercise, the decrease in NKG2A expression suggests a shift towards a more systemic mature NK cell phenotype, consistent with previous findings (34). Interestingly, NKG2A expression and frequency was not altered in uNK cells, however, a trend was visible towards an increase of NKG2A expression by CD56^bright^CD16^-^ NK cells.

In this study, we collected paired PB and MB samples from the same menstrual cycle for each participant to compare the impact of exercise in systemic and uterine immune environment. Interestingly, the changes in the function and receptor expression of NK cells were confined to pNK cells and were not detected in uNK cells. It is well-established that pNK cells and uNK cells are distinct cell subsets (12), albeit the origin of uNK cells remains uncertain— some studies suggest that uNK cells originate from recruitment of CD34^-^CD117^+^CD94^-^ NK precursor cells from the PB to the uterus (53–55), while others propose that these cells directly derive from CD34^+^ precursor cells present in the uterus (56, 57). This distinction could raise relevant questions about the diverse responsiveness of systemic versus tissue resident cells to exercise. Some studies have shown that pNK cells are functionally responsive to exercise (58–60). To the best of our knowledge, this is the first study to report effects of exercise on uterine NK cells. Therefore, in the future, it would be interesting to further investigate the differential responses of pNK and uNK cells to exercise.

The effect of exercise on immune cell numbers and function can vary significantly depending on the type, intensity, and duration of the exercise (60–63). Previous studies have reported increased leukocyte mobilization, particularly of NK cells, immediately after acute or high-intensity exercise (61, 62, 64, 65). However, we did not observe an increase in NK cells frequencies which might be attributed to a different duration and the choice for a moderate intensity exercise regimen. Additionally, it was shown that immune cell numbers typically peak shortly after an exercise session and later return to baseline levels (38, 65). Since we did not collect blood immediately after the final training session, the timing of our sample collection may explain the differences in our findings. Interestingly, we observed that the frequencies of immune cells varied between women with uRPL. Some exhibited increased NK cell percentages, while others showed reduced percentages, suggesting that the effects of exercise may differ between individuals. This variability could possibly be attributed to the lifestyle of women prior to the intervention. Previously, it has been shown that people with a sedentary lifestyle exhibited higher C-reactive protein and IL-6 levels, which could indicate low-grade inflammation (66, 67). Moreover, it has been shown that age can also contribute to differential immune responses to exercise. A study from de Almeida-Neto et al. showed that men of older chronological age (34.6 ± 8.3) versus males of a younger age (21.8 ± 1.8) responded differently to exercise in their T cell compartment (68). This highlights, that even within a narrow age-range, immune responses to exercise can differ. In our study, we included women with different lifestyles and ages (between 28 and 40 years old). Therefore, it would be relevant to study possible associations between, among others, prior lifestyle and chronological age in relation to the response of immune cells to an exercise intervention in more detail in the future.

It is important to highlight several limitations that may have influenced the study’s conclusions. The first limitation is that we do not know if the changes induced by exercise are specific for uRPL patients. It may be that these changes also occur in women with uncomplicated pregnancies or, for pNK cells, also in age-matched men. Inclusion of a control group with non-uRPL women or males would be needed to further elucidate this. Second, there are no reference values for the markers that were affected by exercise. Furthermore, we were not able to evaluate multiple consecutive samples to determine the sample variation over time in individual patients by taking samples over multiple menstrual cycles or by sampling an intervention control group with uRPL that did not undergo the exercise intervention. For those markers, it would be interesting to include a larger cohort that could help confirm whether the changes we observe are purely exercise-mediated or whether additional factors may be involved as well. Some studies have already investigated the effects of the menstrual cycle on the expression of different uterine and peripheral NK cell receptors, including KIRs and NKG2A. Both studies showed that the expression was stable over different menstrual cycles (69, 70). Another point of attention is that there could have been a selection bias. It has been suggested that the endometrium of women with uRPL may be less selective for embryo quality, allowing low-quality embryos to implant (71, 72). Since these embryos may fail to develop properly, miscarriage is more likely to occur. In this study, participants were allowed to conceive during the intervention, leading to a high drop-out rate primarily due to pregnancy. As a result, we not only ended up with a relatively small sample size, but we also lost a subgroup of women who may have a higher propensity to conceive easily. As a follow up it would be interesting to perform more in-depth analysis of NK cell phenotypes or function in subgroups of women for example based on number of miscarriages, age, lifestyle factors. In the current study, our groups were underpowered to do this type of analysis. Finally, we were not able yet to correlate immune changes observed in response to the intervention with pregnancy outcomes due to the short follow-up of our study, currently limiting our ability to directly link these findings to clinical benefit. Despite these limitations, our intervention strategy may offer broader health benefits for women with uRPL, as regular physical activity is known to reduce risk factors commonly associated with RPL, such as metabolic syndrome and cardiovascular disease.

In conclusion, our study showed systemic effects of moderate-intensity aerobic exercise on immune phenotype and function in women with uRPL. Although it is difficult to translate these results into clinical significance, this research has offered new insights into the differential impact of exercise on both systemic pNK and tissue-resident uNK cells. Future research should focus on increasing the sample size, including a control cohort, and correlating the pregnancy outcomes to the exercise-dependent changes to identify the clinical relevance of these immune changes in pregnancy success or failure in women with uRPL.

## Data Availability

The raw data supporting the conclusions of this article will be made available by the authors, without undue reservation.
