# Supplementary material for: Impact of a moderate-intensity aerobic exercise intervention on systemic and uterine natural killer cells in women with unexplained recurrent pregnancy loss
Source: Front Immunol. 2025 Jun 4;16:1602939. doi: 10.3389/fimmu.2025.1602939 (PMC12174435; doi:10.3389/fimmu.2025.1602939)
Supplement: Supplementary file 1 [file DataSheet1.pdf]

# Supplementary files

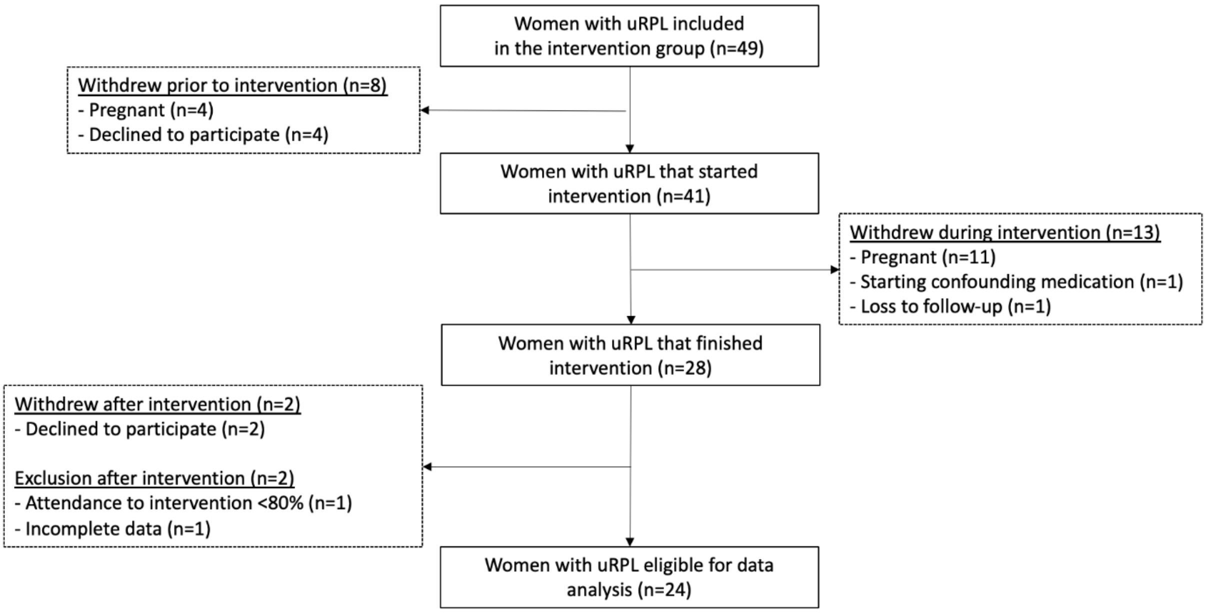


**Supplementary Figure 1. Flow chart of study population**

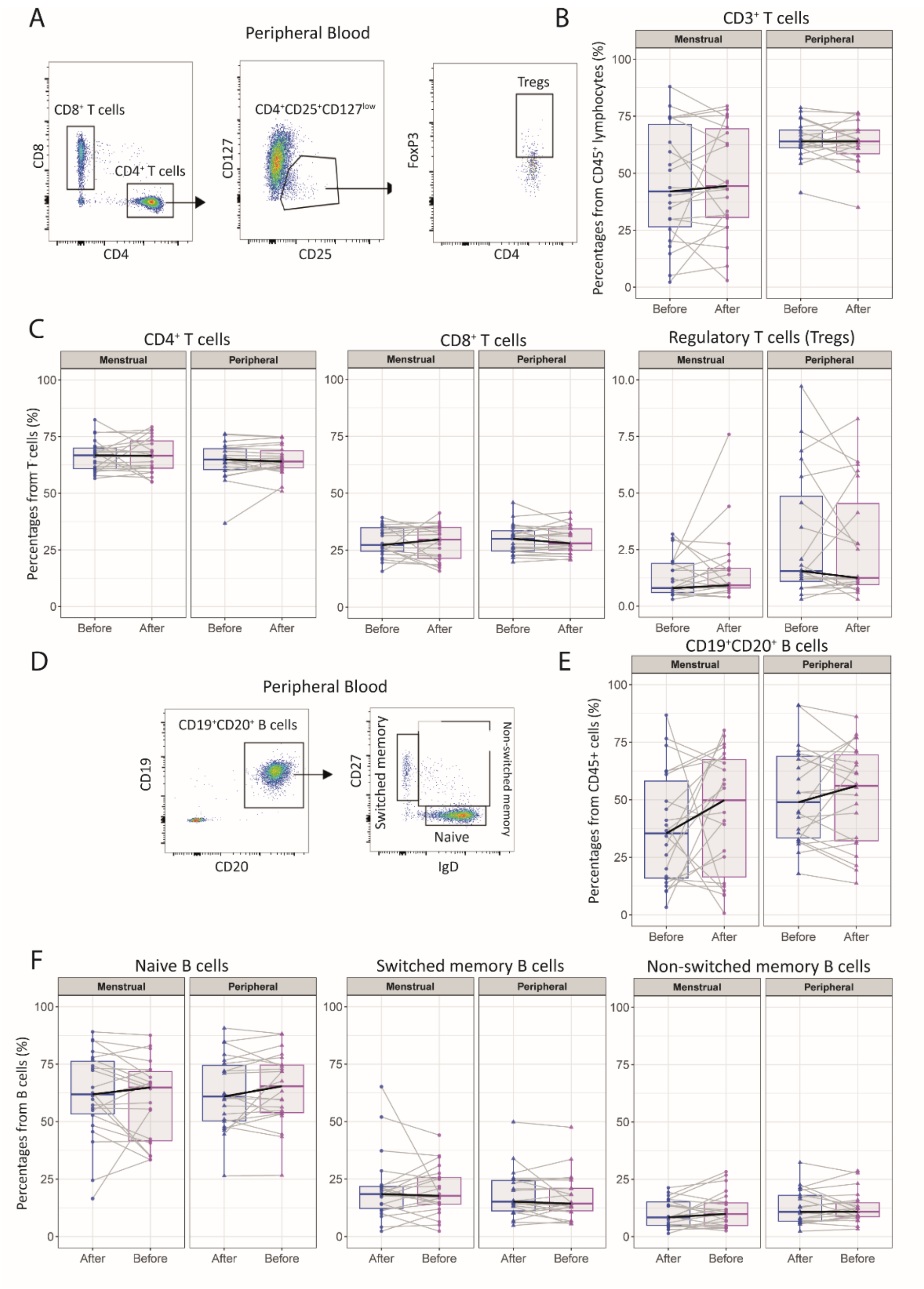


**Supplementary Figure 2. Frequencies of different T cell and B cell subsets in MB and PB before and after the moderate-intensity aerobic exercise intervention in women with uRPL.** A) Representative dot plots showing the gating strategy for determining T cell subsets. B) Percentages of CD56$^{-}$CD3$^{+}$ T cells of CD45$^{+}$ lymphocytes in MB and PB before and after the moderate-intensity aerobic exercise intervention. C) Percentages of CD4$^{+}$ T cells, CD8$^{+}$ T cells, and CD125$^{Low}$CD25$^{+}$ regulatory T cells. D) Representative dot plots showing the gating strategy for determining B cell subsets. E) Percentages of CD19$^{+}$CD20$^{+}$ B cells of CD45$^{+}$ lymphocytes in MB and PB. F) Percentages of naïve B cells, switched memory B cells, and non-switched memory B cells of CD19$^{+}$CD20$^{+}$ B cells. PB n=22, MB n=22. The dots represent the uterine NK cells, the triangles the peripheral NK cells (blue=before intervention, magenta=after intervention). Boxplots visualize the median (bold black line) and the interquartile ranges. A Paired Wilcoxon signed-rank test was performed to determine statistical significance.

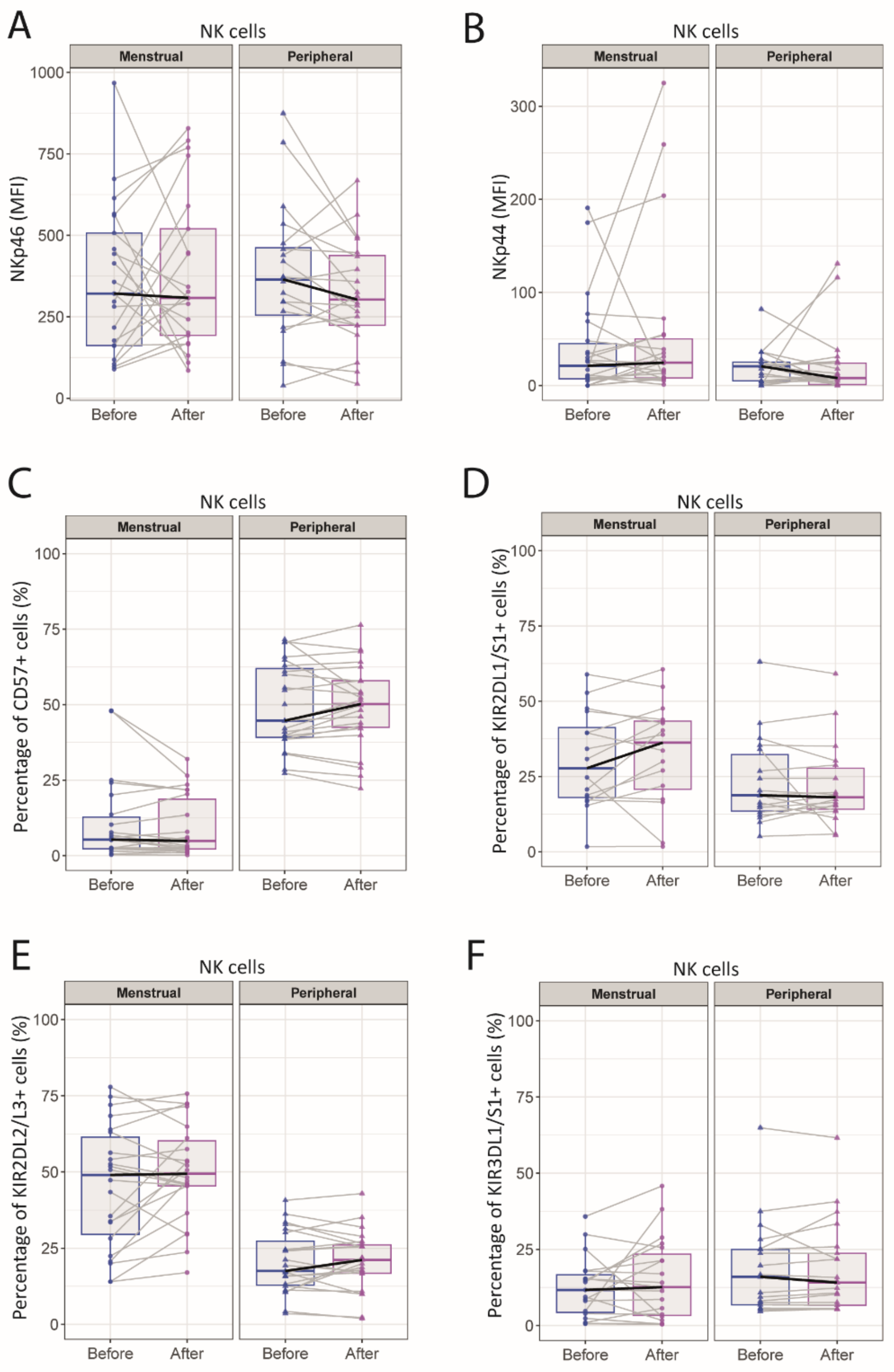


**Supplementary Figure 3. Expression of NKp46, NKp30, CD57, and killer immunoglobulin-like receptors by uterine NK and peripheral NK cells before and after the moderate-intensity aerobic exercise intervention in women with uRPL.** Median fluorescent intensity (MFI) of A) NKp46 and B) NKp44 by NK cells in MB and PB before and after the moderate-intensity aerobic exercise intervention. Percentages of C) $CD57^{+}$, D) $KIR2DL1^{+}$, E) $KIR2DL2/L3^{+}$, and F) $KIR3DL1^{+}$ NK cells in MB and PB. PB n=21, MB n=22. MFIs were normalized to the MFI of the fluorescence minus one control (FMO). The dots represent the uterine NK cells, the triangles the peripheral NK cells (blue=before intervention, magenta=after intervention). Boxplots visualize the median (bold black line) and the interquartile ranges. A Paired Wilcoxon signed-rank test was performed to determine statistical significance.

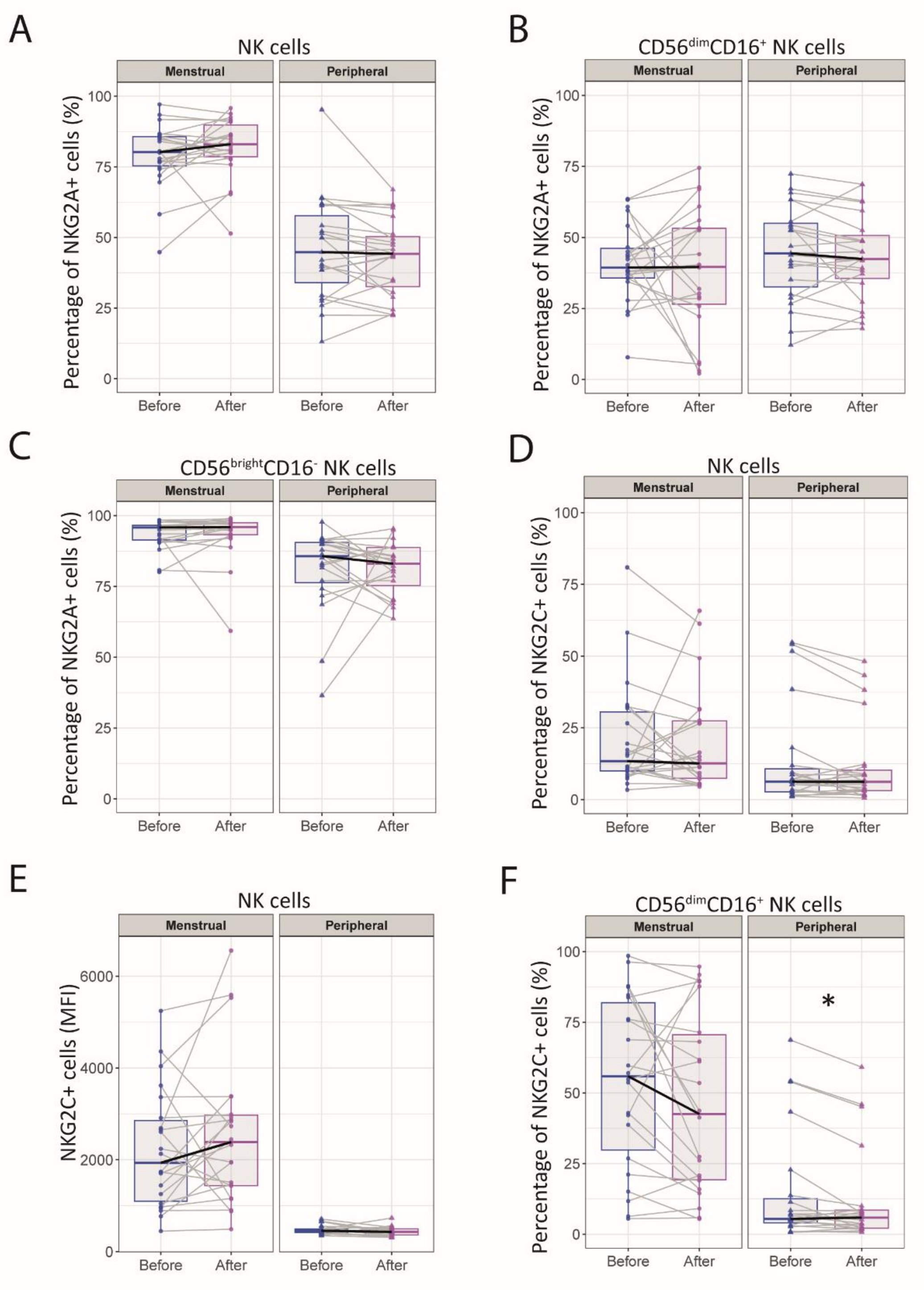


**Supplementary Figure 4. Expression of NKG2A and NKG2C by uterine NK and peripheral NK cells before and after the moderate-intensity aerobic exercise intervention in women with uRPL.** Percentages of NKG2A$^+$ A) CD56$^+$CD3$^-$ total NK cells, B) CD56$^{dim}$CD16$^-$ NK cells, and C) CD56$^{bright}$CD16$^-$ NK cells in MB and PB before and after the moderate-intensity aerobic exercise intervention. D) Percentages of NKG2C$^+$CD56$^+$CD3$^-$ total NK cells. E) Median fluorescent intensity (MFI) of NKG2C in total NK cells. F) Percentage of NKG2C$^+$CD56$^{dim}$CD16$^-$ NK cells in MB and PB. PB n=23, MB n=22. The dots represent the uterine NK cells, the triangles the peripheral NK cells (blue=before intervention, magenta=after intervention). Boxplots visualize the median (bold black line) and the interquartile ranges. MFIs were normalized to the MFI of the fluorescent minus one (FMO) control. A Paired Wilcoxon signed-rank test was performed to determine statistical significance (*$p<0.05$).

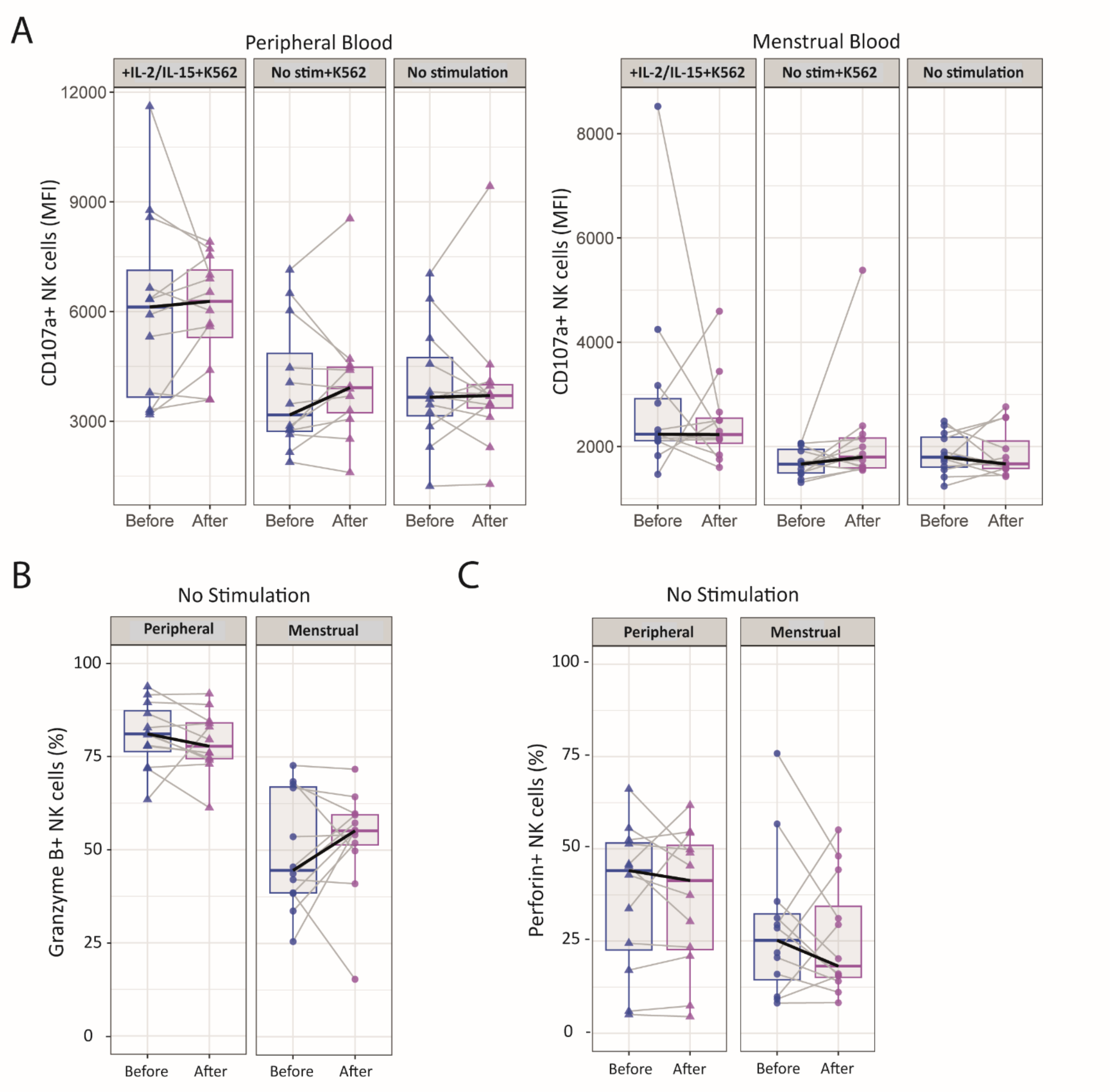


**Supplementary Figure 5. Expression of functional makers CD107a, granzyme-B, and perforin by uterine NK and peripheral NK cells before and after the moderate-intensity aerobic exercise intervention in women with uRPL.** A) Median Fluorescent Intensity (MFI) of $CD107a^{+}$ NK cells from MB and PB in the IL-2 and IL-15 stimulated and co-cultured with K562 (IL-2/IL-15+K562, left panel), unstimulated and co-cultured with K562 (No stim+K562, central panel), and unstimulated (No stimulation, right panel) conditions. B) Percentages of granzyme $B^{+}$ unstimulated NK cells in PB and MB. C) PB n=12, MB, n=12. Percentages of $perforin^{+}$ unstimulated NK cells in PB and MB. MFIs were normalized to the MFI of the fluorescence minus one (FMO) control. The dots represent the uterine NK cells, the triangles the peripheral NK cells (blue=before intervention, magenta=after intervention). Boxplots visualize the median (bold black line) and the interquartile ranges. A Paired Wilcoxon signed-rank test was performed to determine statistical significance.

**Supplementary Table 1. Exclusion criteria**

| **Exclusion criteria** |
|---|
| Age >40 years |
| BMI >40 |
| Current use of immunosuppressive or biological drugs |
| Current use of hormonal contraceptives |
| HIV positivity |
| Current or recent (<2 weeks) symptomatic genital infection |
| Pre-existent diabetes mellitus, autoimmune disease, or overt cardiovascular disease |
| Vaccinations within 1 month prior to or during sampling and intervention |
| Breastfeeding |
| Current or recent (<3 months ago) pregnancy |
| (Physical) inabilities to follow the moderate-intensity aerobe cycling training |
| Participants who are not capable of signing the informed consent |

**Supplementary Table 2. List of antibodies used in flow cytometry experiments**

| Antibody | Fluorochrome | Clone |
|---|---|---|
| CD45 | Krome Orange | J33 |
| CD3 | BV605 | HIT3a |
| CD3 | PE-Cy7 | UCHT1 |
| CD56 | BV711 | NCAM16.2 |
| CD16 (FcγRII) | APC-H7 | 3G8 |
| CD4 | PE-Cy7 | SFCI12T4D1 |
| CD8 | APC-AF700 | B9.11 |
| CD19 | APC-R700 | HIB19 |
| CD20 | BV786 | 2H7 |
| HLA-DR | FITC | G46-6 |
| CD38 | PE | HB.7 |
| IgD | APC | IA6-2 |
| CD27 | BV421 | M-T271 |
| CD127 | AF647 | eBioRDR5 |
| CD197 (CCR7) | BV421 | G043H7 |
| FoxP3 | PE | PCH101 |
| CD45RA | FITC | ALB11 |
| CD25 | PerCP-Cy5.5 | M-A251 |
| CD158a (KIR2DL1) | FITC | REA284 |
| CD159c (NKG2C) | AF700 | 134522 |
| CD336 (NKp44) | PE | Z231 |
| CD337 (NKp30) | PerCP-Cy5.5 | P30-15 |
| CD335 (NKp46) | PE-Cy7 | BAB281 |
| CD161 (KLRB1) | eFluor 450 | HP-3G10 |
| Perforin | Pacific Blue | dG9 |
| Granzyme B | PerCP-Cy5.5 | QA16A02 |
| IFN-γ | PE-Cy7 | 4S.B3 |
| CD107a | PE | H4A3 |
| CD16 | AF700 | 3G8 |
